# *Announcement*: National Infertility Awareness Week, April 23–29, 2017

**DOI:** 10.15585/mmwr.mm6615a5

**Published:** 2017-04-21

April 23–29 is National Infertility Awareness Week and is intended to increase awareness of infertility, which affects the reproductive systems of both women and men (*1*). In general, infertility is defined as the inability of couples to achieve pregnancy after ≥1 year of trying (*1*). However, given that fertility in women is known to decline steadily with age, some providers evaluate and treat women aged ≥35 years after 6 months of intercourse without the use of contraception (*1*). Causes of infertility include genetic abnormalities, certain acute and chronic diseases, exposure to certain environmental toxins, smoking, and excessive alcohol use (*2*).

During 2011–2013, approximately 1.6 million (6%) married women aged 15–44 years in the United States reported difficulty getting pregnant (*3*). Approximately 4 million (9%) men aged 25–44 years reported that they or their partner had consulted a doctor for advice, testing, or treatment for infertility during their lifetime (*4*). Infertility might contribute to stress, anxiety, and depression in couples trying to conceive, and treatment can be medically invasive and expensive. In addition, fertility treatments can be associated with health problems for women and resulting children (*2*), especially those related to the increased risk for multiple gestation.

In collaboration with partners, CDC developed the *National Public Health Action Plan for the Detection, Prevention, and Management of Infertility*, which identifies opportunities to prevent and reduce infertility and improve outcomes for couples undergoing fertility treatment. Additional information regarding infertility is available at https://www.cdc.gov/reproductivehealth/Infertility/index.htm. Information on National Infertility Awareness Week is available at http://www.resolve.org/national-infertility-awareness-week/home-page.html.
